# *N*-(4-Bromo­butano­yl)-*N*′-phen­ylthio­urea

**DOI:** 10.1107/S1600536811021684

**Published:** 2011-06-11

**Authors:** Bohari M. Yamin, Nur Eliyanti Ali Othman

**Affiliations:** a School of Chemical Sciences and Food Technology, Universiti Kebangsaan Malaysia, UKM 43600 Bangi Selangor, Malaysia.

## Abstract

The asymmetric unit of the title compound, C_11_H_13_Br_1_N_2_O_1_S_1_, consists of two independent mol­ecules, which are linked by N—H⋯O hydrogen bonds, forming a dimer. Both mol­ecules maintain the *trans-*-*cis* configuration with respect to the position of the butanoyl groups and benzene rings against the thiono group across the C—N bonds. The mol­ecule is stabilized by intra­molecular N—H⋯O hydrogen bonds. Inter­molecular N—H⋯S, C—H⋯S and C—H⋯π inter­actions also occur.

## Related literature

For related structures of halocarbonyl thio­urea derivatives, see: Othman *et al.* (2010 ▶); Yamin *et al.* (2011 ▶). For standard bond lengths, see: Allen *et al.* (1987 ▶).
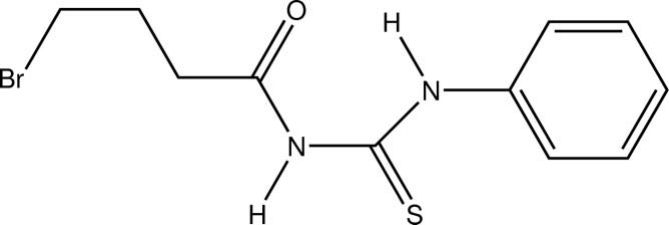


## Experimental

### 

#### Crystal data

C_11_H_13_BrN_2_OS
*M* *_r_* = 301.20
Monoclinic, 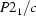

*a* = 14.689 (3) Å
*b* = 10.349 (2) Å
*c* = 18.249 (4) Å
β = 111.220 (5)°
*V* = 2586.0 (10) Å^3^
*Z* = 8
Mo *K*α radiation
μ = 3.32 mm^−1^
*T* = 298 K
0.50 × 0.33 × 0.10 mm

#### Data collection

Bruker SMART APEX CCD area-detector diffractometer
Absorption correction: multi-scan (*SADABS*; Bruker, 2000 ▶) *T*_min_ = 0.287, *T*_max_ = 0.732
15722 measured reflections
5077 independent reflections
3076 reflections with *I* > 2/s(*I*)
*R*_int_ = 0.048

#### Refinement

*R*[*F* ^2^ > 2σ(*F* ^2^)] = 0.058
*wR*(*F* ^2^) = 0.177
*S* = 1.01
5077 reflections
289 parameters
H-atom parameters constrained
Δρ_max_ = 0.89 e Å^−3^
Δρ_min_ = −0.75 e Å^−3^

### 

Data collection: *SMART* (Bruker, 2000 ▶); cell refinement: *SAINT* (Bruker, 2000 ▶); data reduction: *SAINT*; program(s) used to solve structure: *SHELXS97* (Sheldrick, 2008 ▶); program(s) used to refine structure: *SHELXL97* (Sheldrick, 2008 ▶); molecular graphics: *SHELXTL* (Sheldrick, 2008 ▶); software used to prepare material for publication: *SHELXTL*, *PARST* (Nardelli, 1995 ▶) and *PLATON* (Spek, 2009) ▶.

## Supplementary Material

Crystal structure: contains datablock(s) global, I. DOI: 10.1107/S1600536811021684/dn2691sup1.cif

Structure factors: contains datablock(s) I. DOI: 10.1107/S1600536811021684/dn2691Isup2.hkl

Supplementary material file. DOI: 10.1107/S1600536811021684/dn2691Isup3.cml

Additional supplementary materials:  crystallographic information; 3D view; checkCIF report

## Figures and Tables

**Table 1:** Hydrogen-bond geometry (Å, °) *Cg*1 and *Cg*2 are the centroids of the C6–C11 and C17–C22 rings, respectively.

*D*—H⋯*A*	*D*—H	H⋯*A*	*D*⋯*A*	*D*—H⋯*A*
N2—H2⋯O1	0.86	2.02	2.690 (6)	134
N4—H4⋯O2	0.86	2.03	2.687 (6)	133
N2—H2⋯O2	0.86	2.41	3.140 (5)	143
N4—H4⋯O1	0.86	2.33	3.049 (6)	142
N1—H1⋯S2^i^	0.86	2.53	3.386 (4)	173
C14—H14*A*⋯S2^ii^	0.97	2.78	3.711 (6)	160
N3—H3⋯S1^iii^	0.86	2.59	3.445 (4)	176
C2—H2*A*⋯*Cg*2	0.97	2.69	3.405 (8)	131
C13—H13*A*⋯*Cg*1	0.97	2.83	3.708 (6)	150

Symmetry codes: (i) 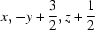; (ii) 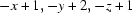; (iii) 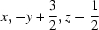.

**Fig. 1.**
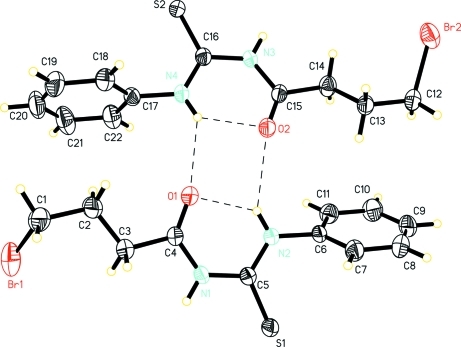
The molecular structure of (I) with the atom labeling scheme. Displacement ellipsods are drawn at the 50% probability level. H atoms are represented as small spheres of arbitrary radii. Hydrogen bonds are shown as dashed lines.

**Fig. 2.**
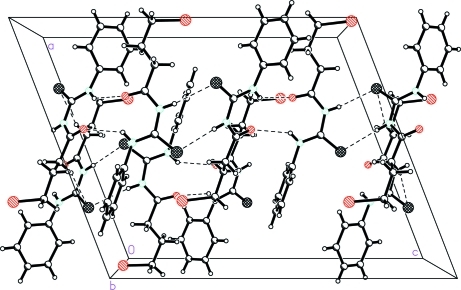
Packing diagram.

## supplementary crystallographic information

### Comment

The title compound (I) is similar to previously reported *N*-(4-chlorobutanoyl)-*N*'-phenyl thiourea (Yamin *et al.*, 2011), except the chlorine atom is replaced by bromine atom. The asymmetric unit also consists of two independent molecules linked by N-H···O hydrogen bonds forming a pseudo dimer (Fig. 1).

Both molecules are not planar. The thiourea fragments (C4/N1/C5/S1/N2/C6), (C15/N3/C16/S2/N4/C17) and the benzene rings, (C6—C11) and (C17—C22) are each planar with maximum deviation of 0.055 (4)Å for N3 atom from the least square plane. The dihedral angles between the benzene ring and thiourea fragment in each molecule are 71.9 (2)° and 82.2 (3)° respectively and comparable to those in the *N*-(4-chlorobutanoyl)-*N*'-phenyl thiourea (72.98 (10)°, 81.47 (14)°). Both molecules maintain the *trans*-*cis* configuration with respect to the butanoyl and benzene ring against the thiono group across their C—N bonds respectively.

There are intramolecular hydrogen bonds, N2—H2···O1, N4—H4···O2 and C3—H3A···Br1 forming two pseudo-six membered rings, [O1···H2/N2/C5/N1/C4], [O2···H4/N4/C16/N3/C15] and a pseudo-five membered rings, [Br1···H3A/C3/C2/C1] respectively. In the crystal structure, the molecules are linked by N1—H1···S2, N3—H3···S1 and N4—H4···O1 intermolecular hydrogen bonds (symmetry codes as in Table 1) to form trimers which are then liked by the N4—H4···O1 and N2—H2···O2 intramolecular hydrogen bonds (Table 1). In addition, there are also C2—H2A..π and C13—H13A..π bonds with the centeroid benzene rings *Cg*2 (C17—C22)and *Cg*1 (C6—C11)respectively. All these interactions build up a complicated three dimensional network (Table 1).

### Experimental

30 ml acetone solution of aniline (1.33 g, 14 mmol) was added into 30 ml acetone containing 4-bromobutanoyl chloride (2.60 g, 14 mmol) and ammonium thiocyanate (1.09 g, 14 mmol). The mixture was refluxed for 2 h. The solution was filtered and left to evaporate at room temperature. Colourless crytals were obtained after two days of slow evaporation. Yield 90%; m.p 392.3–393.2 K.

### Refinement

All H atoms attached to C and N atoms were fixed geometrically and treated as riding with C—H= 0.93–0.97 Å(aromatic and methylene) and N—H= 0.86 Å(amino) with *U*_iso_(H)=1.2*U*_eq_(C or N). There are highest peak 1.26Å and deepest hole 0.93Å for Br1 atom.

### Crystal data

**Table:**

C_11_H_13_BrN_2_OS	*F*(000) = 1216
*M**_r_* = 301.20	*D*_x_ = 1.547 Mg m^−^^3^
Monoclinic, *P*2_1_/*c*	Melting point = 392.3–393.2 K
Hall symbol: -P 2ybc	Mo *K*α radiation, λ = 0.71073 Å
*a* = 14.689 (3) Å	Cell parameters from 2780 reflections
*b* = 10.349 (2) Å	θ = 1.5–26.0°
*c* = 18.249 (4) Å	µ = 3.32 mm^−^^1^
β = 111.220 (5)°	*T* = 298 K
*V* = 2586.0 (10) Å^3^	Block, colourless
*Z* = 8	0.50 × 0.33 × 0.10 mm

### Data collection

**Table:**

Bruker SMART APEX CCD area-detector diffractometer	5077 independent reflections
Radiation source: fine-focus sealed tube	3076 reflections with *I* > 2/s(*I*)
graphite	*R*_int_ = 0.048
Detector resolution: 83.66 pixels mm^-1^	θ_max_ = 26.0°, θ_min_ = 1.5°
ω scan	*h* = −17→18
Absorption correction: multi-scan (*SADABS*; Bruker, 2000)	*k* = −12→9
*T*_min_ = 0.287, *T*_max_ = 0.732	*l* = −21→22
15722 measured reflections	

### Refinement

**Table:**

Refinement on *F*^2^	Primary atom site location: structure-invariant direct methods
Least-squares matrix: full	Secondary atom site location: difference Fourier map
*R*[*F*^2^ > 2σ(*F*^2^)] = 0.058	Hydrogen site location: inferred from neighbouring sites
*wR*(*F*^2^) = 0.177	H-atom parameters constrained
*S* = 1.01	*w* = 1/[σ^2^(*F*_o_^2^) + (0.0843*P*)^2^ + 2.7585*P*] where *P* = (*F*_o_^2^ + 2*F*_c_^2^)/3
5077 reflections	(Δ/σ)_max_ = 0.001
289 parameters	Δρ_max_ = 0.89 e Å^−^^3^
0 restraints	Δρ_min_ = −0.75 e Å^−^^3^

### Special details

**Table:**

Geometry. All e.s.d.'s (except the e.s.d. in the dihedral angle between two l.s. planes) are estimated using the full covariance matrix. The cell e.s.d.'s are taken into account individually in the estimation of e.s.d.'s in distances, angles and torsion angles; correlations between e.s.d.'s in cell parameters are only used when they are defined by crystal symmetry. An approximate (isotropic) treatment of cell e.s.d.'s is used for estimating e.s.d.'s involving l.s. planes.
Refinement. Refinement of *F*^2^ against ALL reflections. The weighted *R*-factor *wR* and goodness of fit *S* are based on *F*^2^, conventional *R*-factors *R* are based on *F*, with *F* set to zero for negative *F*^2^. The threshold expression of *F*^2^ > σ(*F*^2^) is used only for calculating *R*-factors(gt) *etc*. and is not relevant to the choice of reflections for refinement. *R*-factors based on *F*^2^ are statistically about twice as large as those based on *F*, and *R*- factors based on ALL data will be even larger.

### Fractional atomic coordinates and isotropic or equivalent isotropic displacement parameters (Å^2^)

**Table:**

	*x*	*y*	*z*	*U*_iso_*/*U*_eq_	
Br1	1.00981 (7)	0.72786 (12)	1.00605 (6)	0.1327 (4)	
Br2	0.28595 (5)	0.69236 (7)	0.28367 (4)	0.0784 (3)	
S1	0.48380 (9)	0.47455 (14)	0.83458 (8)	0.0528 (4)	
S2	0.74841 (9)	1.02020 (13)	0.52423 (8)	0.0513 (3)	
O1	0.7046 (3)	0.6521 (4)	0.7416 (2)	0.0615 (10)	
O2	0.5743 (3)	0.6931 (3)	0.5762 (2)	0.0555 (9)	
N1	0.6510 (3)	0.5574 (4)	0.8311 (2)	0.0458 (10)	
H1	0.6702	0.5361	0.8799	0.055*	
N2	0.5208 (3)	0.5717 (4)	0.7131 (2)	0.0478 (10)	
H2	0.5610	0.6068	0.6946	0.057*	
N3	0.6000 (3)	0.8661 (4)	0.5094 (2)	0.0427 (9)	
H3	0.5736	0.9093	0.4666	0.051*	
N4	0.7312 (3)	0.8488 (4)	0.6266 (2)	0.0491 (10)	
H4	0.6998	0.7869	0.6382	0.059*	
C1	1.0005 (4)	0.6481 (8)	0.9059 (4)	0.091 (2)	
H1A	1.0458	0.6906	0.8861	0.109*	
H1B	1.0193	0.5579	0.9148	0.109*	
C2	0.8995 (4)	0.6569 (7)	0.8455 (4)	0.0767 (19)	
H2A	0.8814	0.7474	0.8370	0.092*	
H2B	0.9003	0.6225	0.7963	0.092*	
C3	0.8241 (4)	0.5886 (6)	0.8656 (3)	0.0588 (14)	
H3A	0.8255	0.6194	0.9162	0.071*	
H3B	0.8399	0.4972	0.8709	0.071*	
C4	0.7220 (4)	0.6051 (5)	0.8060 (3)	0.0486 (12)	
C5	0.5530 (3)	0.5391 (4)	0.7884 (3)	0.0407 (11)	
C6	0.4217 (3)	0.5511 (5)	0.6608 (3)	0.0443 (11)	
C7	0.3889 (4)	0.4292 (6)	0.6373 (3)	0.0623 (15)	
H7	0.4297	0.3584	0.6562	0.075*	
C8	0.2944 (5)	0.4120 (7)	0.5851 (4)	0.0777 (19)	
H8	0.2713	0.3292	0.5688	0.093*	
C9	0.2350 (4)	0.5158 (8)	0.5576 (4)	0.080 (2)	
H9	0.1712	0.5036	0.5230	0.096*	
C10	0.2687 (4)	0.6376 (7)	0.5803 (4)	0.0740 (18)	
H10	0.2281	0.7084	0.5609	0.089*	
C11	0.3631 (4)	0.6559 (6)	0.6324 (3)	0.0576 (14)	
H11	0.3866	0.7388	0.6479	0.069*	
C12	0.2997 (4)	0.6205 (6)	0.3859 (3)	0.0624 (15)	
H12A	0.2589	0.6690	0.4076	0.075*	
H12B	0.2772	0.5317	0.3792	0.075*	
C13	0.4040 (4)	0.6247 (5)	0.4427 (3)	0.0510 (12)	
H13A	0.4079	0.5829	0.4914	0.061*	
H13B	0.4447	0.5762	0.4208	0.061*	
C14	0.4432 (4)	0.7593 (5)	0.4606 (3)	0.0544 (13)	
H14A	0.3995	0.8094	0.4785	0.065*	
H14B	0.4437	0.7986	0.4124	0.065*	
C15	0.5449 (3)	0.7663 (5)	0.5219 (3)	0.0442 (11)	
C16	0.6924 (3)	0.9049 (4)	0.5572 (3)	0.0423 (11)	
C17	0.8234 (4)	0.8868 (5)	0.6832 (3)	0.0497 (12)	
C18	0.9079 (4)	0.8368 (7)	0.6801 (4)	0.0709 (17)	
H18	0.9066	0.7786	0.6409	0.085*	
C19	0.9966 (5)	0.8749 (9)	0.7373 (5)	0.093 (2)	
H19	1.0549	0.8426	0.7356	0.112*	
C20	0.9985 (6)	0.9573 (9)	0.7942 (5)	0.100 (3)	
H20	1.0579	0.9810	0.8321	0.120*	
C21	0.9142 (6)	1.0065 (7)	0.7972 (5)	0.095 (2)	
H21	0.9164	1.0643	0.8368	0.114*	
C22	0.8253 (5)	0.9712 (6)	0.7417 (4)	0.0694 (16)	
H22	0.7675	1.0042	0.7439	0.083*	

### Atomic displacement parameters (Å^2^)

**Table:**

	*U*^11^	*U*^22^	*U*^33^	*U*^12^	*U*^13^	*U*^23^
Br1	0.0786 (6)	0.1732 (10)	0.1102 (7)	−0.0418 (6)	−0.0091 (5)	−0.0171 (6)
Br2	0.0770 (5)	0.0753 (5)	0.0611 (4)	0.0005 (3)	−0.0013 (3)	−0.0011 (3)
S1	0.0424 (7)	0.0709 (9)	0.0440 (7)	−0.0065 (6)	0.0144 (6)	0.0010 (6)
S2	0.0425 (7)	0.0632 (8)	0.0445 (7)	−0.0108 (6)	0.0111 (6)	0.0055 (6)
O1	0.044 (2)	0.079 (3)	0.053 (2)	−0.0140 (18)	0.0074 (17)	0.0128 (19)
O2	0.049 (2)	0.054 (2)	0.051 (2)	−0.0114 (17)	0.0036 (17)	0.0105 (17)
N1	0.036 (2)	0.060 (3)	0.036 (2)	−0.0008 (18)	0.0054 (18)	0.0018 (18)
N2	0.038 (2)	0.060 (3)	0.040 (2)	−0.0077 (19)	0.0073 (18)	0.0072 (19)
N3	0.035 (2)	0.052 (2)	0.035 (2)	−0.0029 (18)	0.0048 (17)	0.0031 (17)
N4	0.036 (2)	0.059 (3)	0.043 (2)	−0.0082 (19)	0.0032 (18)	0.0071 (19)
C1	0.041 (3)	0.132 (6)	0.096 (5)	0.011 (4)	0.021 (4)	0.041 (5)
C2	0.041 (3)	0.121 (6)	0.063 (4)	0.005 (3)	0.013 (3)	0.024 (4)
C3	0.046 (3)	0.070 (4)	0.052 (3)	−0.007 (3)	0.008 (3)	0.006 (3)
C4	0.041 (3)	0.057 (3)	0.043 (3)	−0.008 (2)	0.009 (2)	−0.002 (2)
C5	0.041 (3)	0.039 (3)	0.040 (3)	0.002 (2)	0.012 (2)	−0.002 (2)
C6	0.034 (2)	0.058 (3)	0.036 (2)	−0.006 (2)	0.007 (2)	0.001 (2)
C7	0.057 (3)	0.058 (3)	0.060 (3)	−0.006 (3)	0.006 (3)	0.006 (3)
C8	0.068 (4)	0.080 (5)	0.071 (4)	−0.030 (4)	0.008 (3)	−0.003 (3)
C9	0.039 (3)	0.125 (6)	0.059 (4)	−0.017 (4)	−0.002 (3)	0.012 (4)
C10	0.042 (3)	0.091 (5)	0.079 (4)	0.010 (3)	0.010 (3)	0.015 (4)
C11	0.051 (3)	0.059 (3)	0.061 (3)	0.003 (3)	0.018 (3)	0.001 (3)
C12	0.051 (3)	0.066 (4)	0.061 (3)	−0.017 (3)	0.010 (3)	−0.005 (3)
C13	0.044 (3)	0.051 (3)	0.051 (3)	−0.007 (2)	0.009 (2)	0.000 (2)
C14	0.044 (3)	0.048 (3)	0.060 (3)	0.001 (2)	0.006 (3)	0.004 (2)
C15	0.039 (3)	0.045 (3)	0.046 (3)	−0.006 (2)	0.013 (2)	−0.005 (2)
C16	0.038 (3)	0.046 (3)	0.042 (3)	0.001 (2)	0.012 (2)	−0.004 (2)
C17	0.043 (3)	0.051 (3)	0.045 (3)	−0.004 (2)	0.004 (2)	0.012 (2)
C18	0.047 (3)	0.096 (5)	0.065 (4)	−0.002 (3)	0.014 (3)	0.007 (3)
C19	0.043 (4)	0.128 (7)	0.095 (6)	−0.002 (4)	0.008 (4)	0.031 (5)
C20	0.068 (5)	0.108 (6)	0.084 (6)	−0.030 (5)	−0.020 (4)	0.015 (5)
C21	0.090 (6)	0.077 (5)	0.080 (5)	−0.014 (4)	−0.014 (4)	−0.008 (4)
C22	0.065 (4)	0.057 (4)	0.068 (4)	−0.002 (3)	0.002 (3)	−0.001 (3)

### Geometric parameters (Å, °)

**Table:**

Br1—C1	1.964 (8)	C7—C8	1.383 (8)
Br2—C12	1.950 (6)	C7—H7	0.9300
S1—C5	1.676 (5)	C8—C9	1.360 (9)
S2—C16	1.680 (5)	C8—H8	0.9300
O1—C4	1.211 (6)	C9—C10	1.363 (9)
O2—C15	1.196 (6)	C9—H9	0.9300
N1—C4	1.373 (6)	C10—C11	1.382 (8)
N1—C5	1.380 (6)	C10—H10	0.9300
N1—H1	0.8600	C11—H11	0.9300
N2—C5	1.325 (6)	C12—C13	1.508 (7)
N2—C6	1.437 (6)	C12—H12A	0.9700
N2—H2	0.8600	C12—H12B	0.9700
N3—C16	1.381 (6)	C13—C14	1.497 (7)
N3—C15	1.381 (6)	C13—H13A	0.9700
N3—H3	0.8600	C13—H13B	0.9700
N4—C16	1.321 (6)	C14—C15	1.510 (7)
N4—C17	1.429 (6)	C14—H14A	0.9700
N4—H4	0.8600	C14—H14B	0.9700
C1—C2	1.497 (8)	C17—C18	1.364 (8)
C1—H1A	0.9700	C17—C22	1.372 (8)
C1—H1B	0.9700	C18—C19	1.399 (9)
C2—C3	1.467 (8)	C18—H18	0.9300
C2—H2A	0.9700	C19—C20	1.337 (12)
C2—H2B	0.9700	C19—H19	0.9300
C3—C4	1.510 (7)	C20—C21	1.357 (11)
C3—H3A	0.9700	C20—H20	0.9300
C3—H3B	0.9700	C21—C22	1.381 (9)
C6—C7	1.362 (7)	C21—H21	0.9300
C6—C11	1.365 (7)	C22—H22	0.9300
			
C4—N1—C5	128.6 (4)	C9—C10—C11	120.0 (6)
C4—N1—H1	115.7	C9—C10—H10	120.0
C5—N1—H1	115.7	C11—C10—H10	120.0
C5—N2—C6	123.1 (4)	C6—C11—C10	119.4 (6)
C5—N2—H2	118.4	C6—C11—H11	120.3
C6—N2—H2	118.4	C10—C11—H11	120.3
C16—N3—C15	127.8 (4)	C13—C12—Br2	112.0 (4)
C16—N3—H3	116.1	C13—C12—H12A	109.2
C15—N3—H3	116.1	Br2—C12—H12A	109.2
C16—N4—C17	122.6 (4)	C13—C12—H12B	109.2
C16—N4—H4	118.7	Br2—C12—H12B	109.2
C17—N4—H4	118.7	H12A—C12—H12B	107.9
C2—C1—Br1	112.1 (5)	C14—C13—C12	113.0 (4)
C2—C1—H1A	109.2	C14—C13—H13A	109.0
Br1—C1—H1A	109.2	C12—C13—H13A	109.0
C2—C1—H1B	109.2	C14—C13—H13B	109.0
Br1—C1—H1B	109.2	C12—C13—H13B	109.0
H1A—C1—H1B	107.9	H13A—C13—H13B	107.8
C3—C2—C1	115.0 (5)	C13—C14—C15	113.9 (4)
C3—C2—H2A	108.5	C13—C14—H14A	108.8
C1—C2—H2A	108.5	C15—C14—H14A	108.8
C3—C2—H2B	108.5	C13—C14—H14B	108.8
C1—C2—H2B	108.5	C15—C14—H14B	108.8
H2A—C2—H2B	107.5	H14A—C14—H14B	107.7
C2—C3—C4	114.1 (5)	O2—C15—N3	123.6 (4)
C2—C3—H3A	108.7	O2—C15—C14	123.2 (4)
C4—C3—H3A	108.7	N3—C15—C14	113.2 (4)
C2—C3—H3B	108.7	N4—C16—N3	117.6 (4)
C4—C3—H3B	108.7	N4—C16—S2	123.9 (4)
H3A—C3—H3B	107.6	N3—C16—S2	118.5 (3)
O1—C4—N1	123.3 (5)	C18—C17—C22	120.7 (5)
O1—C4—C3	123.4 (5)	C18—C17—N4	120.3 (5)
N1—C4—C3	113.3 (4)	C22—C17—N4	118.9 (5)
N2—C5—N1	117.3 (4)	C17—C18—C19	118.6 (7)
N2—C5—S1	124.6 (4)	C17—C18—H18	120.7
N1—C5—S1	118.0 (3)	C19—C18—H18	120.7
C7—C6—C11	120.9 (5)	C20—C19—C18	120.7 (7)
C7—C6—N2	120.2 (5)	C20—C19—H19	119.7
C11—C6—N2	118.8 (5)	C18—C19—H19	119.7
C6—C7—C8	119.3 (6)	C19—C20—C21	120.5 (7)
C6—C7—H7	120.4	C19—C20—H20	119.7
C8—C7—H7	120.4	C21—C20—H20	119.7
C9—C8—C7	120.2 (6)	C20—C21—C22	120.4 (7)
C9—C8—H8	119.9	C20—C21—H21	119.8
C7—C8—H8	119.9	C22—C21—H21	119.8
C8—C9—C10	120.3 (6)	C17—C22—C21	119.1 (7)
C8—C9—H9	119.8	C17—C22—H22	120.5
C10—C9—H9	119.8	C21—C22—H22	120.5
			
Br1—C1—C2—C3	−62.8 (8)	Br2—C12—C13—C14	62.5 (6)
C1—C2—C3—C4	176.7 (6)	C12—C13—C14—C15	175.7 (5)
C5—N1—C4—O1	8.4 (8)	C16—N3—C15—O2	−4.7 (8)
C5—N1—C4—C3	−169.5 (5)	C16—N3—C15—C14	174.8 (4)
C2—C3—C4—O1	11.4 (8)	C13—C14—C15—O2	−33.8 (7)
C2—C3—C4—N1	−170.7 (5)	C13—C14—C15—N3	146.7 (5)
C6—N2—C5—N1	176.4 (4)	C17—N4—C16—N3	−175.7 (4)
C6—N2—C5—S1	−2.2 (7)	C17—N4—C16—S2	4.1 (7)
C4—N1—C5—N2	−0.5 (7)	C15—N3—C16—N4	−6.3 (7)
C4—N1—C5—S1	178.3 (4)	C15—N3—C16—S2	173.9 (4)
C5—N2—C6—C7	−71.4 (6)	C16—N4—C17—C18	−85.2 (6)
C5—N2—C6—C11	111.9 (5)	C16—N4—C17—C22	96.6 (6)
C11—C6—C7—C8	−1.3 (8)	C22—C17—C18—C19	−1.0 (9)
N2—C6—C7—C8	−178.0 (5)	N4—C17—C18—C19	−179.0 (5)
C6—C7—C8—C9	0.1 (9)	C17—C18—C19—C20	0.9 (11)
C7—C8—C9—C10	0.9 (10)	C18—C19—C20—C21	−0.7 (12)
C8—C9—C10—C11	−0.8 (10)	C19—C20—C21—C22	0.5 (12)
C7—C6—C11—C10	1.4 (8)	C18—C17—C22—C21	0.8 (9)
N2—C6—C11—C10	178.1 (5)	N4—C17—C22—C21	178.9 (5)
C9—C10—C11—C6	−0.3 (9)	C20—C21—C22—C17	−0.6 (11)

### Hydrogen-bond geometry (Å, °)

**Table:**

Cg1 and Cg2 are the centroids of the C6–C11 and C17–C22 rings, respectively.

**Table:**

*D*—H···*A*	*D*—H	H···*A*	*D*···*A*	*D*—H···*A*
N2—H2···O1	0.86	2.02	2.690 (6)	134
N4—H4···O2	0.86	2.03	2.687 (6)	133
C3—H3A···Br1	0.97	2.84	3.321 (6)	112
C14—H14B···Br2	0.97	2.86	3.293 (5)	108
N2—H2···O2	0.86	2.41	3.140 (5)	143
N4—H4···O1	0.86	2.33	3.049 (6)	142
N1—H1···S2^i^	0.86	2.53	3.386 (4)	173
C14—H14A···S2^ii^	0.97	2.78	3.711 (6)	160
N3—H3···S1^iii^	0.86	2.59	3.445 (4)	176
C2—H2A···Cg2	0.97	2.69	3.405 (8)	131
C13—H13A···Cg1	0.97	2.83	3.708 (6)	150

Symmetry codes:  (i) *x*, −*y*+3/2, *z*+1/2; (ii) −*x*+1, −*y*+2, −*z*+1; (iii) *x*, −*y*+3/2, *z*−1/2.
